# An automated method for stem diameter measurement based on laser module and deep learning

**DOI:** 10.1186/s13007-023-01045-7

**Published:** 2023-07-05

**Authors:** Sheng Wang, Rao Li, Huan Li, Xiaowen Ma, Qiang Ji, Fu Xu, Hongping Fu

**Affiliations:** 1 School of Information Science and Technology, Beijing Forestry University, Beijing, 100083 China; 2 Engineering Research Center for Forestry-oriented Intelligent Information Processing, National Forestry and Grassland Administration, Beijing, 100083 China; 3 Space Star Technology Co., Ltd., Beijing, 100048 China

**Keywords:** Measurement, Forest inventory, Laser module, Image sensor, Deep learning

## Abstract

**Background:**

Measuring stem diameter (SD) is a crucial foundation for forest resource management, but current methods require expert personnel and are time-consuming and costly. In this study, we proposed a novel device and method for automatic SD measurement using an image sensor and a laser module. Firstly, the laser module generated a spot on the tree stem that could be used as reference information for measuring SD. Secondly, an end-to-end model was performed to identify the trunk contour in the panchromatic image from the image sensor. Finally, SD was calculated from the linear relationship between the trunk contour and the spot diameter in pixels.

**Results:**

We conducted SD measurements in three natural scenarios with different land cover types: transitional woodland/shrub, mixed forest, and green urban area. The SD values varied from 2.00 cm to 89.00 cm across these scenarios. Compared with the field tape measurements, the SD data measured by our method showed high consistency in different natural scenarios. The absolute mean error was 0.36 cm and the root mean square error was 0.45 cm. Our integrated device is low cost, portable, and without the assistance of a tripod. Compared to most studies, our method demonstrated better versatility and exhibited higher performance.

**Conclusion:**

Our method achieved the automatic, efficient and accurate measurement of SD in natural scenarios. In the future, the device will be further explored to be integrated into autonomous mobile robots for more scenarios.

## Background

Stem Diameter (SD) is a key parameter for estimating standing timber volume [1], assessing economic value [2], and planning silvicultural interventions [3]. Larger trees are normally measured using the diameter at breast height (DBH) [4, 5], whereas for trees below breast height including multi-stemmed trees [6], shrubs [7] and saplings [8], measurements are generally taken below the most common location where the stem section forms multiple leaders [5, 9]. In such vegetation, the traditional methods of measuring SD require trained personnel to determine the location and angle of the measurement. Automatic and cost-effective methods of measuring have become much-needed tools for forest inventories.

The main challenge in automatically measuring DBH of individual tree largely lies in the limitations of conventional measurement methods. In earlier studies, foresters manually measured trees using altimeter, tape measure, and diameter tape [10–12]. These methods are relatively reliable and low-cost, but they are also time-consuming, labor-intensive, and error-prone [13–16]. Other studies have compared and evaluated different manual measurement methods, such as the angle gauge [17], Bitterlich sector fork [18, 19], electronic tree measuring fork [20] and Bilt-more stick [21]. The measurement method based on projection geometry improves the efficiency. However, the accuracy of the device depends on the forester experience, and additional auxiliary tools are required to indicate altitude [10, 22]. Similarly, some methods were designed based on optical measurement principles such as optical calipers [23, 24], optical forks [25]. The measurement uncertainty increases with DBH. The inaccessibility of the target area and the limitations of the device accuracy constrain the application of above methods.

Some semi-automatic methods based on light detection and ranging (LiDAR) have gained popularity recently. Terrestrial laser scanning (TLS) is proved to be a promising solution for deriving DBH from TLS data through either direct geometric fitting or tree stem modeling and separation [26–29]. The advantage of TLS data is that it can capture forest data in detail and enable time series analysis. However, the device is high-cost, complex data processing and demands high expertise [30]. Backpack and vehicle-based LiDAR systems are spatially flexible [31, 32], but vehicle-based LiDAR is constrained by difficult terrain and available roads, while the stability of backpack LiDAR system is affected by irregular movement. Despite the acceptable accuracy of LiDAR-based methods, automation of these methods still faces the challenges of cost of hardware, complexity of data processing, and portability.

To achieve high automation in acquiring tree structure information, close-range photography technology and a segmentation and fitting algorithm based on point cloud data are widely applied. However, some challenges still exist. The automated methods to generate dense point clouds for estimating DBH using close-range photography are attempted, but are susceptible to light conditions [33–35]. Gao et al. [29] modeled the forest based on structure from motion photogrammetry to automatically estimate DBH by circular fitting. The method is economical but not applicable to scenarios with deviations in tree and circularity. Machine vision methods can obtain the pixel size of objects from rich image information. Wu et al. [36] used machine vision and close-range photogrammetry to measure the DBH of multiple trees from an image taken by a smartphone. The method is convenient, efficient and has great potential for development to bring to a wide range of users. However, the coordinate system conversion in photogrammetry is very complicated and cannot guarantee the accuracy of transformation from 2D images to 3D coordinates [37]. The tree tilt angle, ground slope and photographic distance limit its use in daily practice.

This paper aims to achieve automated stem diameter measurements by integrating a laser module and an image sensor. It also considers relevant factors to reduce the professional and economic costs of the method. The accuracy of the method is evaluated and analyzed in different scenarios to verify its generality and feasibility.

## Materials and methods

### Device description

**Fig. 1:**
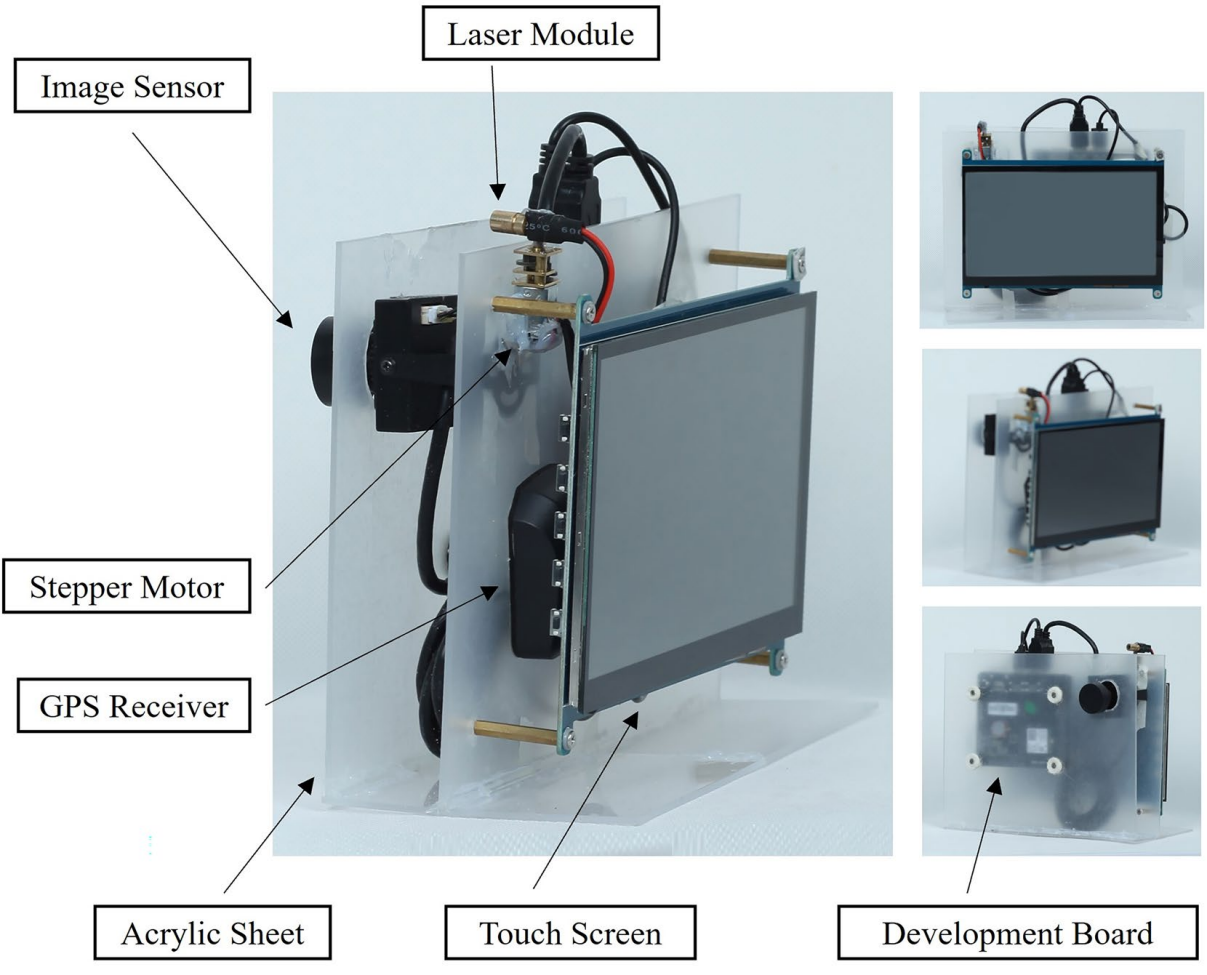
The structure of the developed device, including image sensor, laser module, development board, stepper motor, GPS receiver, touch screen, and acrylic sheet. The battery is embedded internally. The housing of the device is made of acrylic sheet

The device proposed in this paper is used to estimate the stem diameter of trees in the field, including image sensor, laser module, development board, stepper motor, GPS receiver, touch screen, and acrylic board. The image sensor was used to capture panchromatic images of the target tree while the laser module formed a fixed size spot on the tree trunk. Then, analysis module calculated the panchromatic image to obtain the SD. The device is highly integrated, eliminating the need for tripods and other external auxiliary devices (Fig. 1). Table 1 lists the core parameters of the laser module and image sensor used in this device.

The development board has an embedded system module with a GPU and CPU built-in. When the device is running, the embedded system module reads live video from the image sensor interface and displays it on the touch screen. The image sensor and the laser module are coaxial. The horizontal angle between the image sensor and the optical axis of the laser module is fixed. The laser module is situated right above the image sensor and is controlled by a stepper motor that rotates at a modest speed. When the spot emerges on the tree trunk, the operator clicks a touch screen button to capture an image of the target tree. In order to avoid repeated measurements, the GPS receiver is in charge of recording the location of the target tree.

**Table 1:** Core parameters of the device components

Module	Core parameters	Values
Image sensor	Image size	1920 × 1080 pixels
	Focal length	6.00 mm
	Image sensor type	CMOS
	Vertical field of View	60$$^\circ$$
	Infrared filter	980 nm Narrow Band
	Price	$ 32.83
Laser module	Wavelength	980 nm
	Spot size	3.00–30.00 mm
	Output power	300 mw
	Angle of divergence	1.05$$^\circ$$
	Working distance	0.05-30.00 m
	Price	$ 27.07
Development board	CPU	H2 Quad-core Cortex-A7 H.265/HEVC 1080P
	GPU	Mali400MP2 GPU @600MHz
	Memory(SDRAM)	512MB DDR3
	Power supply	5V 3A
	Price	$ 22.21
Stepper motor	Actual rated input voltage	3 V–5 V
	Speed	15 RPM
	Motor size	16.0 mm * 12.0 mm * 9.9 mm
	Price	$ 0.50
GPS receiver	Voltage	5 V
	Size	48.0 mm * 35.0 mm * 13.5 mm
	Receiving mode	GPS,BeiDou
	Price	$ 3.50
Touch screen	Resolution	320*480 DOTS
	Interface	HDMI
	Price	$ 8.26
Acrylic sheet	Thickness	2 mm
	Width	300 mm
	Length	200 mm
	Price	$ 0.56
Battery	Capacity	3000 mA
	Voltage	5 V
	Price	$ 9.07

### Workflow

**Fig. 2:**
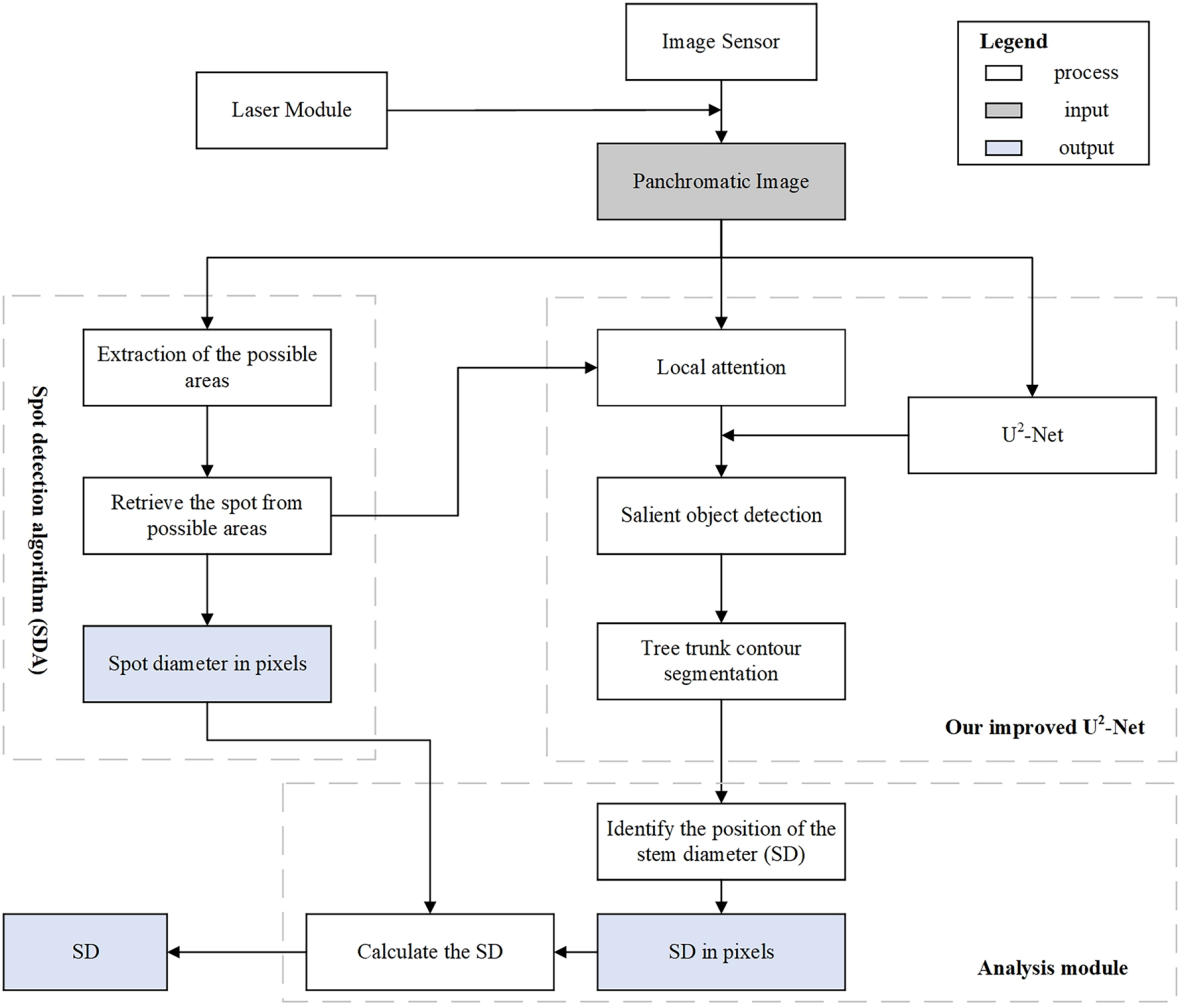
Workflow diagram of the device, including three sub-processes: the spot detection algorithm (SDA), our improved U$^{2}$-Net, and the analysis module

The logical structure of our automated approach consists of three parts (Fig. 2), i.e., the spot detection algorithm (SDA), our improved U$^{2}$-Net, and the analysis module. The SDA provides reference information for the analysis module and key point information for our improved U$^{2}$-Net. Our improved U$^{2}$-Net obtains visual saliency map and segments trunk contour. Finally, the analysis module combines the reference information and the trunk contour information to calculate the SD.

### Algorithms

#### SDA

The SDA is proposed to retrieve the location of the spot centroid and to calculate the number of pixels in the spot diameter. Reducing the impact of lighting noise on the algorithm of panchromatic images is a key issue [38, 39]. The angle $\alpha$ between the image sensor and the laser module also causes the spot to form a slight distortion in original image. Therefore, SDA keeps the image features invariant during processing by constructing a pixel coordinate system, and detects the spot by multi-scale images and circular fitting methods.

**Fig. 3:**
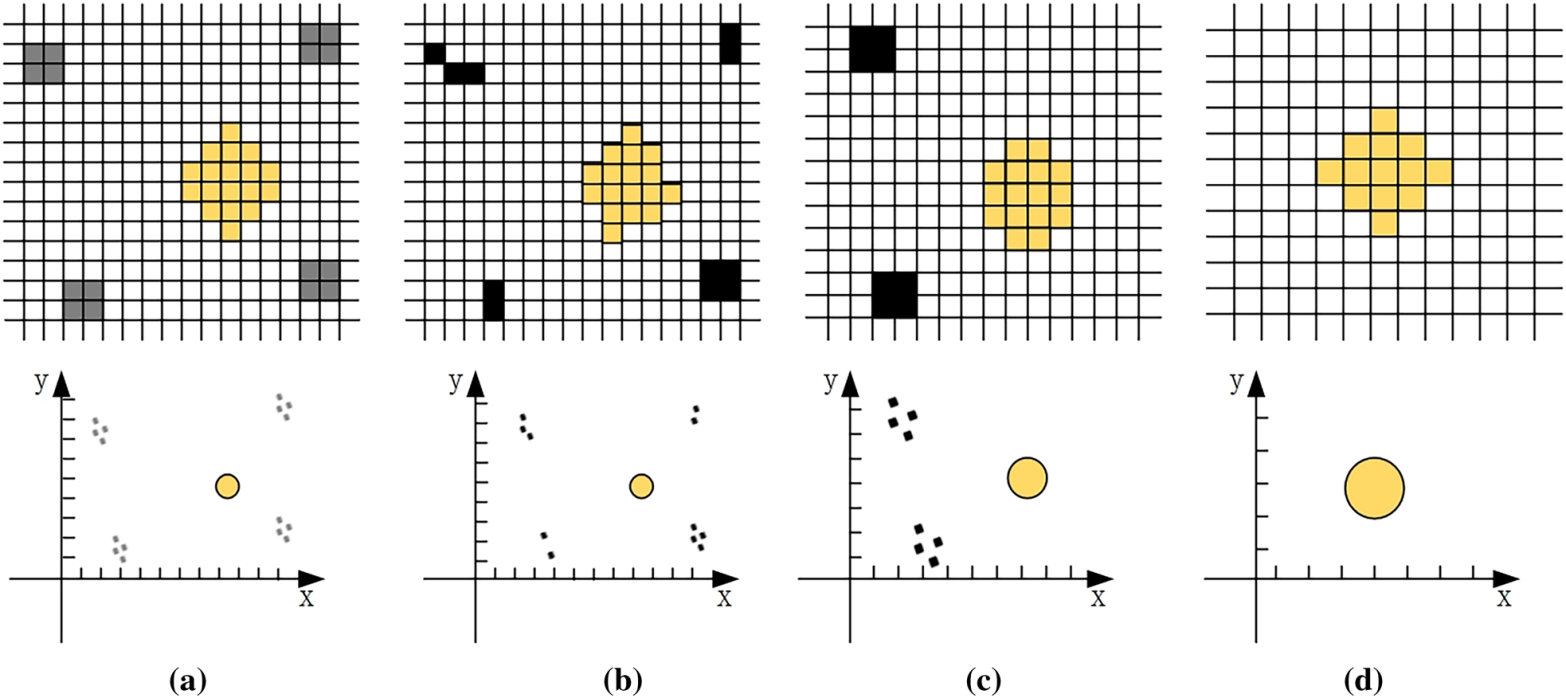
Diagram of circular structural elements in the spot detection algorithm (SDA) and their variation in the coordinate system. The gray is uneven light, the yellow represents the spot and the black is the disturbing factor. **a** The original image and its coordinate system. **b** The original image and coordinate system after opening operation. **c** Cropped image and coordinate system. **d** Spot position image and coordinate system

SDA establishes a pixel coordinate system for the original image, which is composed of black and white regions (Fig. 3a). The gray areas are the uneven lighting conditions, the yellow area is the spot, and the white areas are the backgrounds. We perform an opening operation on the original image. The opening operation is divided into two steps: first, erosion is used to eliminate small blobs. Then, the dilation is used to regenerate the size of the original object. The difference between the original image and the image after the opening operation is represented by the circular structural elements (CSE). The CSE include the spot and the disturbing factors. To remove the disturbing factors close to the image edge, each image is cropped to 15/16 of the original scale, and the output is shown in Fig. 3c. In order to balance the enhancement of the spot with the suppression of disturbing factors, we only iterate on the above steps twice.

The spot is usually an isolated spot after morphological processing and is closest to the center of the image compared to most disturbing factors. Retrieving the spot from the CSE is the key to SDA. The algorithm considered in most of this study is based on the argument of the minimum problem, as shown in Eq. 1.

1 $$\begin{aligned} \mathop {\arg \min }_{x \in S} \ \Vert F(x) \Vert , \end{aligned}$$

where *S* is the centroids of CSE. When the distance between factor *x* and the image centroid is minimum, the minimum value can be obtained in linear space *F*. The cropping operation of the algorithm changes the position of the spot. In order to keep the invariance of the image features, the variable $\lambda$ is introduced in the calculation process for computing the optimal solution $\Omega ^{*}(x)$, as shown in Eq. 2. *C*(*x*) is the centroid coordinate of the image.

2 $$\begin{aligned} \Omega ^{*}(x) := F(x) + \lambda C(x). \end{aligned}$$

The mathematical distribution of grayscale values in the original images was explored in order to separate spot and disturbing factors more precisely, as shown in Eq. 3.

3 $$\begin{aligned} p \sim N(\mu , \sigma ), \end{aligned}$$

where *N* is the number of pixels with pixel value *p*, $\mu$ is the mean, and $\sigma$ is the standard deviation. Pixels of different nature are labled by thresholds during processing. The pixel values *p* greater than the threshold are assigned as $\eta$. Others are set to 0 (Eq. 4).

4 $$\begin{aligned} p =\left\{ \begin{aligned} \eta&,&if \, p > \frac{1}{2} \eta + \mu , \\ 0&,&otherwise. \end{aligned}\right. \end{aligned}$$

The gray value of the spot in the image decreases in a gradient from the centroid to the edge [40, 41]. The SDA selects the pixel points in the region with large variation of light intensity as the composition of linear space $\Gamma (y)$. The least squares method performs circular fitting on the linear space $\Gamma (y)$ of the optimal solution $\Omega ^{*}(x)$ and estimates the pixel size of spot diameter $S'$, as shown in Eq. 5.

5 $$\begin{aligned} S' = \frac{2}{k} \Vert \Gamma (y) - \Omega ^{*}(x) \Vert _{2}. \end{aligned}$$

In Eq. 5, *k* is the number of vectors in linear space $\Gamma (y)$. The larger the *n*, the more accurately the circle is fitted. Therefore, we experimentally obtained the convergence values *n* to make the value of the linear space $\Gamma (y)$ finite (Fig. 3d).

#### Our improved U$^{2}$-Net

Compared to most state-of-the-art networks such as DPNet [42] and RCSB [43], the U$^{2}$-Net has low memory and computational requirements [44]. The U$^{2}$-Net is a two-level nested U-shaped structure that can keep the image features unchanged. The ReSidual U-block (RSU) module at the bottom level is designed to integrate receptive fields at different scales to capture more contextual information at different scales, while the top level ensures depth and reduces computation [44].

**Fig. 4:**
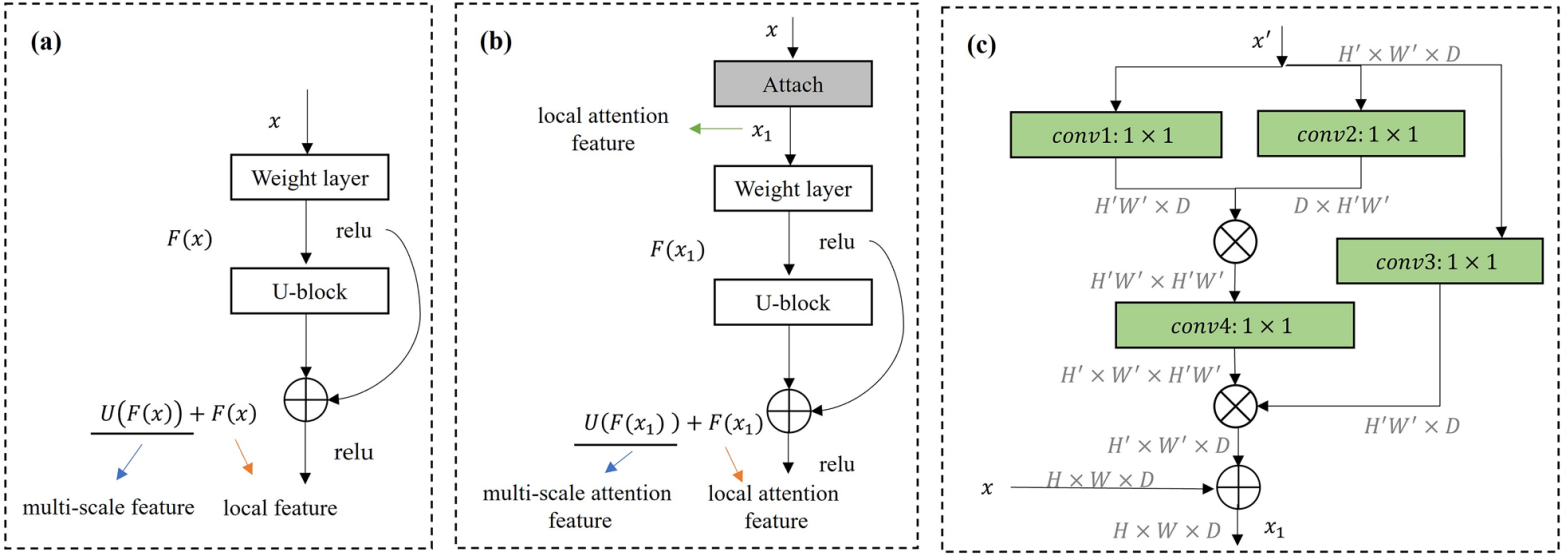
Comparison of the ReSidual U-block (RSU) and our Attach-ReSidual U-block (A-RSU). **a** RSU. **b** A-RSU. **c** Attach

The input feature map *x* is converted into an intermediate map *F*(*x*) in the weight layer. In Fig. 4a, U-block is a U-net-like symmetric encoder-decoder structure that learns to extract and encode the multiscale contextual information *U*(*F*(*x*)) from the intermediate feature map *F*(*x*), where *U* represents a U-net-like structure. The RSU module has a residual connection of multi-scale features and local feature fusion, which can be represented as $U(F(x)) + F(x)$. Due to the complex natural environment and high-resolution images, the larger the receptive field of RSU module is, the richer the local and global features will be. Otherwise, the computational redundancy will be increased. The output features of SDA provide the RSU module with information about the spot. Therefore, we add the *Attach* module, which extracts the local attention feature $x_1$ of input feature map *x* (Fig. 4b). Our Attach-ReSidual U-block (A-RSU) has a novel residual connection which fuses local attention features and the multi-scale attention features by the summation: $U(F(x_1)) + F(x_1)$.

The six stages encoder of U$^{2}$-Net consists of *n* RSU ($n=6$), with different RSU processing feature maps at different spatial resolutions [44]. We extract the area around the spot centroid as local feature map $x^{\prime }$ to be input into the different A-RSU, respectively. The relationship between the local feature map $x^{\prime }$ and the input feature map *x* is shown in Eq. 6. Where *H*, *W* are the height and width of input feature *x*, respectively, and $H^{\prime }_{m}$, $W^{\prime }_{m}$ are the height and width of the *m*-th local feature map $x^{\prime }$.

6 $$\begin{aligned} \frac{H_{m}^{\prime }W_{m}^{\prime }}{HW} = \frac{m}{n} \end{aligned}$$

The structure of the *Attach* module consists of four convolutional layers and the matrix multiplications (Fig. 4c). First, the local feature map $x^{\prime }$ are linearly mapped to obtain three features, and the three features are reshaped. Then, the matrix multiplication is applied to the features output from *conv*1 and *conv*2. The features output from the matrix multiplication are further applied to the $1 \times 1$ convolution to recover the dimensionality of the features. Finally, the result of matrix multiplication of the output from *conv*4 and *conv*3 is added to the input feature map *x*, and then output $x_1$.

**Fig. 5:**
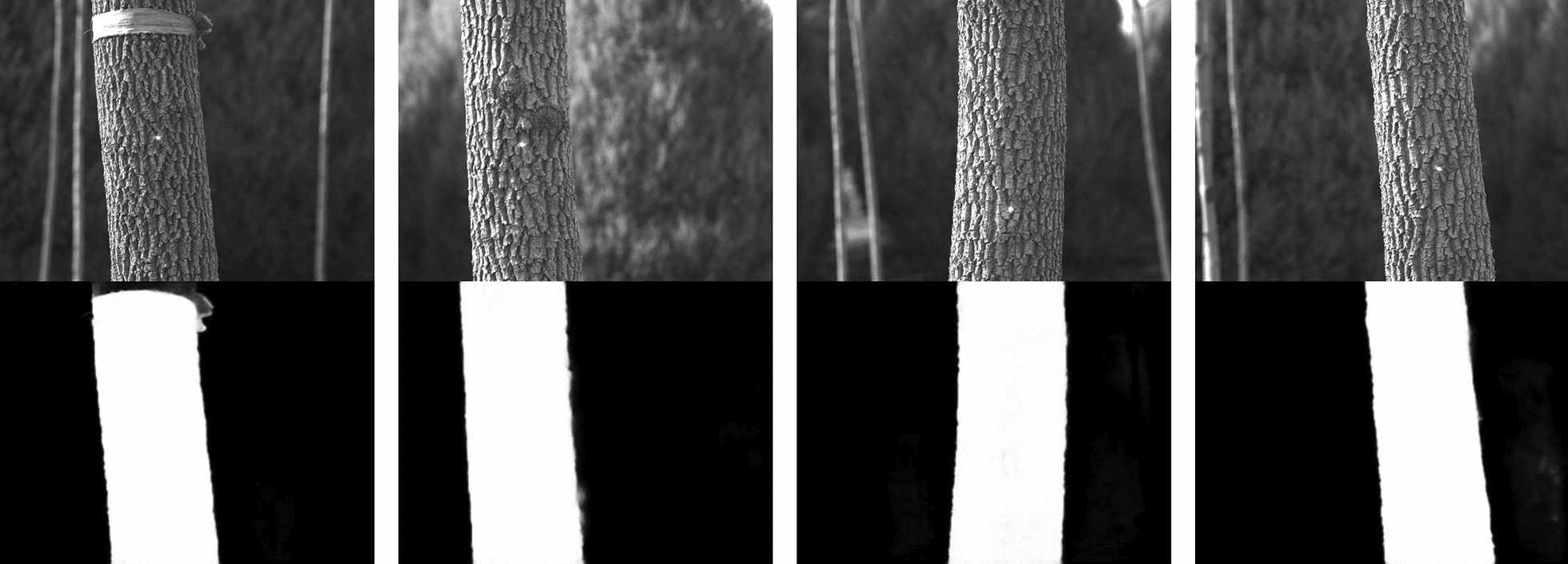
Training and testing of our improved U$^{2}$-Net

We trained our improved U$^{2}$-Net on the trunk dataset of 1600 images created by ourselves, which consisted of 800 panchromatic images and 800 ground truth. The ground truth has the same spatial resolution as the panchromatic images, with either the pixel value of 0 or 255. The 255 indicates the foreground salient object pixels, while the 0 indicates the background pixels (Fig. 5). The Adam optimiser [45] and hyper parameters [44] are set to default values (initial learning rate = $1e-3$, betas = (0.9, 0.999), eps = $1e-8$, weight decay = 0). After 1000 iterations (batch size = 64), the loss function has converged. In the training process, our training loss function is defined as:

7 $$\begin{aligned} L = \sum _{m=1}^{M} \omega ^{m}_{side} l^{m}_{side} \phi ^{m} + \omega ^{m}_{fuse} l^{m}_{fuse} \end{aligned}$$

where $l^{m}_{side}$ is the loss function of the side saliency map and $l^{m}_{fuse}$ is the loss of the final fusion output saliency map of our A-RSU. $\omega ^{m}_{side}$ and $\omega ^{m}_{fuse}$ are the weights of each loss term. In addition, an weight $\phi ^{m}$ is added in this study to improve the adaptability of the loss term. The smaller the resolution of the local feature map $x^{\prime }$, the higher the probability that the *Attach* module captures valid information. The weight $\phi ^{m}$ is determined based on the resolution proportion of the local feature map $x^{\prime }$ and the input feature map *x*.

8 $$\begin{aligned} \phi ^{m} ={\left\{ \begin{array}{ll} 1 - \frac{R(x_m^\prime )}{R(x)}, &{} n < m,\\ 1, &{} n = m \end{array}\right. } \end{aligned}$$

For the case when the local feature map $x^{\prime }$ and the input feature map *x* are the same, we keep the original weights as shown in Eq. 8. $R(x_m^\prime )$ is the resolution of the *m*-th local feature map and *R*(*x*) is the resolution of the input feature map *x*. Finally, the final fused features are used as the output of the model.

#### Analysis module

**Fig. 6:**
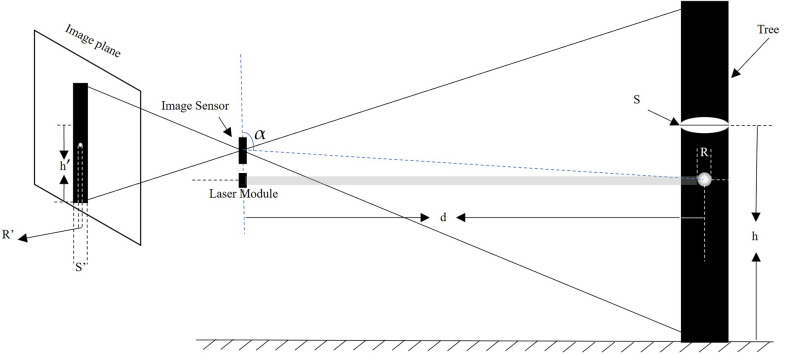
Diagram of the device for SD measurement. The laser module is parallel to the ground, and the image sensor is coaxial with the laser collimator

The analysis module of our method is shown in Fig. 6. The SD is calculated from the tree trunk contour information (object properties, pixel width) extracted by our improved U$^{2}$-Net and spot diameter. *S* is the SD and *R* is the spot diameter. $S'$ and $R'$ are the SD in pixels and spot diameter in pixels mapped to the image, respectively. *d* represents the distance between the device and the target tree. *h* is SD height. The $\alpha$ is the angle formed by the image sensor and the spot. The slight optical distortion caused by $\alpha$ (91$^\circ$) hardly affects the SD calculation.

In the photogrammetry principle and projective geometry theory, the relation of *S*, $S'$, *R* and $R'$ can be expressed as Eq. 9.

9 $$\begin{aligned} \frac{S'}{S} = \frac{R'}{R} \end{aligned}$$

For the measurement of tree DBH, the height in pixels $h'$ can be calculated as in Eq. 10.

10 $$\begin{aligned} \frac{h'}{R'} = \frac{h}{R} \end{aligned}$$

### Experimental data

**Fig. 7:**
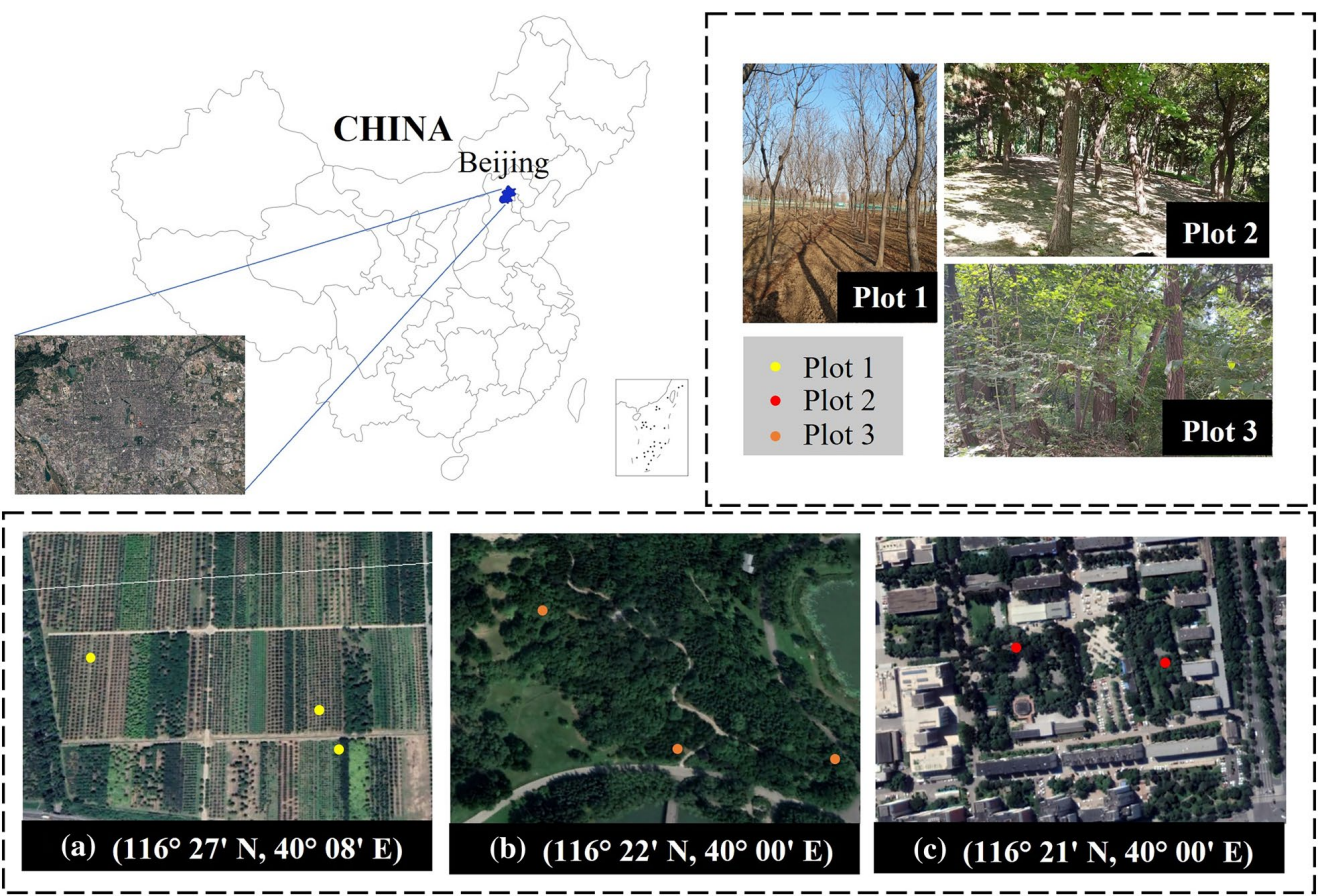
The study area includes three regions in Beijing, China, where blue indicates the provinces. **a**–**c** are the geographical distribution in Google Earth imageries, and Plot 1, Plot 2, and Plot 3 are the field images respectively

The experimental data were primarily obtained from three areas in Beijing, China, i.e., transitional woodland/shrub, mixed forest, and green urban area (Fig. 7). Transitional woodland/shrub is in Changping District ($116^{\circ }27^\prime N$, $40^{\circ } 08^\prime E$), including *Styphnolobium* *japonicum*, *Koelreuteria* *paniculata*, and *Buxus* *sinica*, dominated by saplings and shrubs, with flat terrain. Mixed forest is in Chaoyang District ($116^{\circ } 22^\prime N$, $40^{\circ } 00^\prime E$), including *Ginkgo* *biloba*, *Pinus* *tabuliformis*, and *Acer* *truncatum*, with rugged terrain and difficult to approach in some areas. The green urban area is in Haidian District ($116^{\circ }21^\prime N$, $40^{\circ } 00^\prime E$), including *Robinia* *pseudoacacia* (*L*.) and *Lonicera* *maackii*, dominated by trees with shrubs, with overall flat terrain and undulating in some areas.

**Fig. 8:**
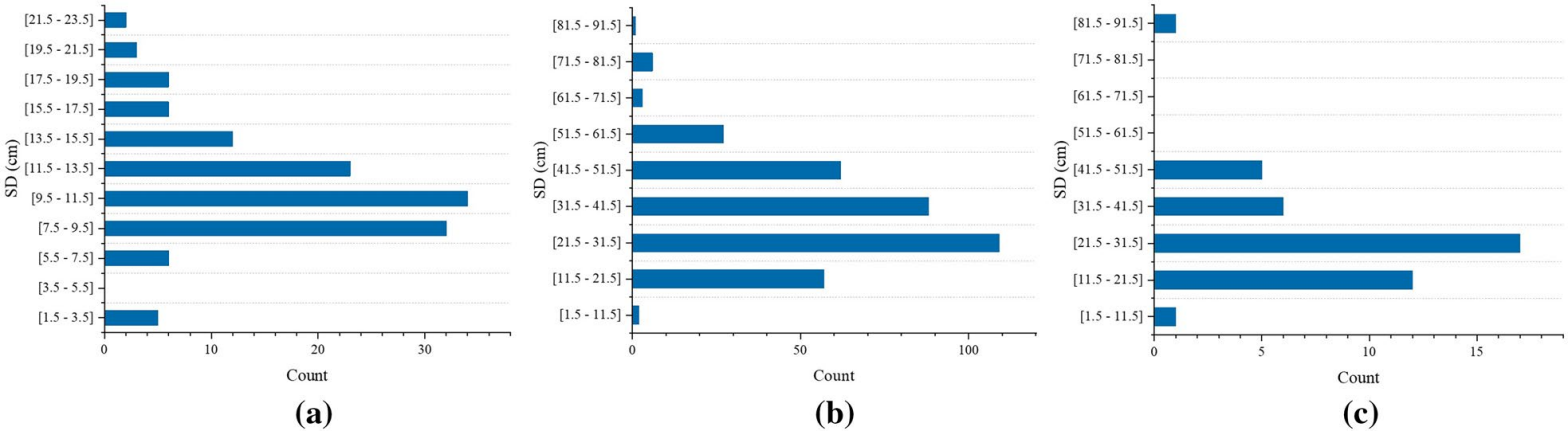
The frequency distribution of stem diameters in field measurements, where (**a**–**c**) correspond to three areas of transitional woodland/shrub, mixed forest, and green urban area, respectively

**Fig. 9:**
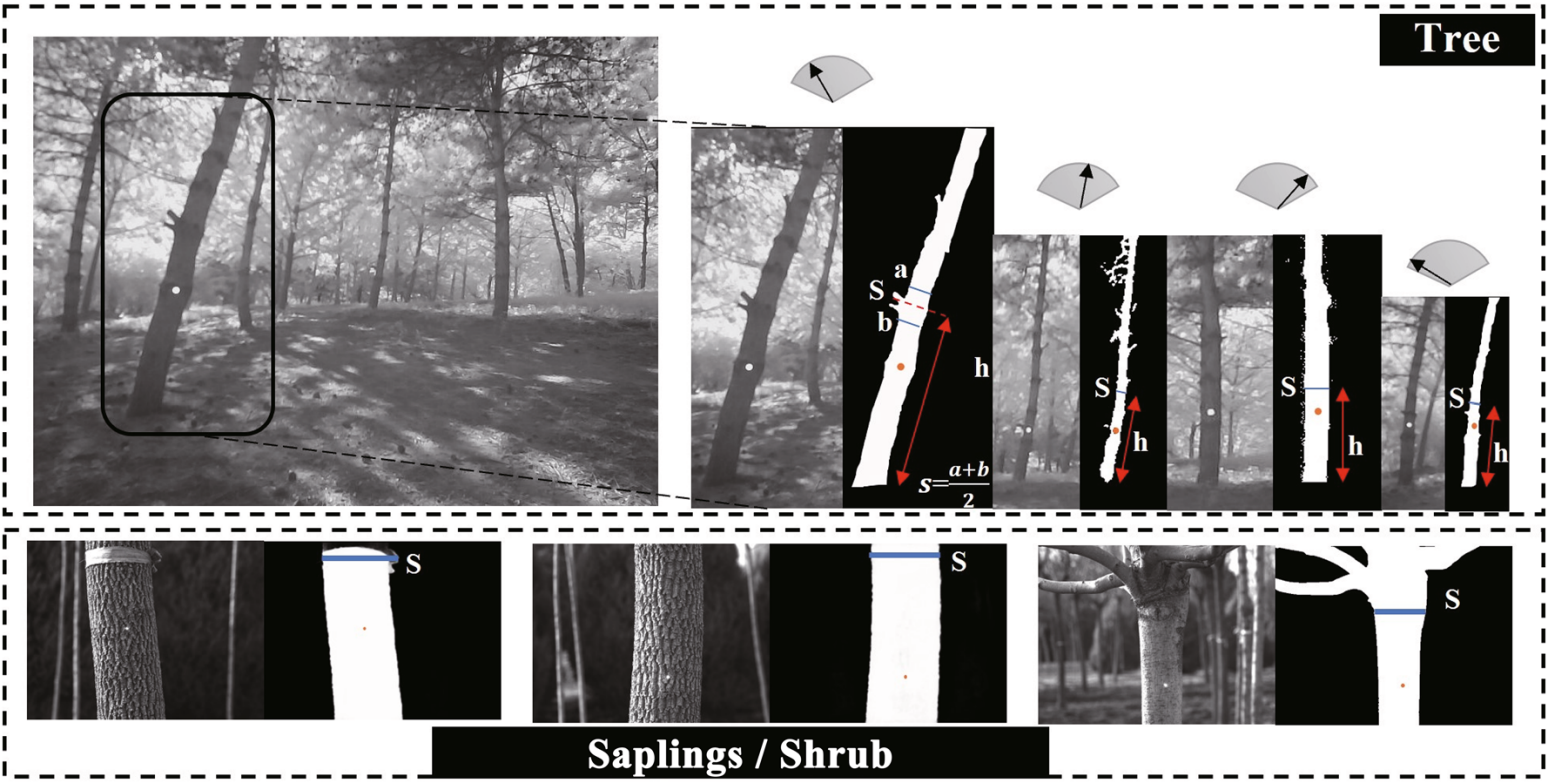
Illustration of measurements of our method for different stem diameters, where the shade on the trunk affects the calculation results. The rotation of the laser module creates visible spots on different trees

In the field measurements, we take tape measure data as reference values to evaluate the accuracy of our method. The distribution of measurement data is shown in Fig. 7. In the transition woodland/shrub, 129 trees were measured, with the largest proportion of trees with SD ranging from 7.50 cm to 15.50 cm. 355 trees were measured in the mixed forest, with a height of 1.30 m and the largest proportion of trees with SD ranging from 11.50 cm to 61.50 cm (Fig. 8). 42 trees were measured in the green urban area, with different types of trees measured at different heights. The largest proportion of SD was between 11.50 cm and 31.50 cm (Fig. 8). The operator keeps the acrylic sheet at the bottom of the device parallel to the ground. The laser module and image sensor are controlled by stepper motors rotating horizontally at a modest speed, allowing multiple trees to be measured at one site (Fig. 9). In transition woodland/shrub, we mark the measurement locations manually on the touch screen. The analysis module in our method calculates the SD of saplings or multi-stemmed trees combining the measurement locations. In mixed forest and green urban area, we do not need to mark measurement locations.

### Evaluation metrics

In this study, the primary interest is to evaluate the performance of our method in different scenarios. For this purpose, different experiments were designed to verify the effectiveness of our method process and results. We introduce structural measurement ($S_{m}$) to evaluate structural similarity [46], enhanced-alignment measurement ($E_{m}$) to evaluate global statistics and local pixel matching information [47], and F-measure ($F_{m}$) to evaluate image-level accuracy. F-measure calculation is shown in Eq. 11. The precision *P* and recall *R* are two metrics widely used in computing to evaluate the quality of results. *P* is the precision of a model, while *R* reflects the completeness of a model. $F_m$ is a weighted summation average of *P* and *R*. $\beta$ represents parameter.

11 $$\begin{aligned} F_m = (1 + \beta ^2) \times \frac{P \times R}{\beta ^2 \times P + R} \end{aligned}$$

In addition, Eq. 12 is introduced to improve the adequacy of evaluation measures for trunk contour images [48], where $\omega$ is the weight.

12 $$\begin{aligned} F_m^\omega = (1 + \beta ^2) \times \frac{P^\omega \times R^\omega }{(\beta ^2 \times P^\omega ) + R^\omega } \end{aligned}$$

To compare the differences between reference and measured values, relative error (*RE*), root mean square error (*RMSE*), mean squared error (*MSE*), mean absolute error (*MAE*), and the coefficient of determination ($R^2$) are calculated by the Eq. 13. The RE is the ratio of the absolute error of the measurement to the actual SD $S_{i}$ multiplied by 100%. The number of samples is *num*. $S_{i}$ denotes the *i*-th calculated SD. $\widehat{S}$ denotes reference values.

13 $$\begin{aligned} {\left\{ \begin{array}{ll} RE = \frac{\mid S_{i} - \widehat{S} \mid }{S_{i}} \times 100 \\ MSE = \frac{1}{num} \sum _{i=1}^{num}(S_{i} - \widehat{S})^{2} \\ RMSE = \sqrt{\frac{\sum _{i=1}^{num}(S_{i} - \widehat{S})^{2}}{n}} \\ MAE = \frac{1}{num} \sum _{i=1}^{num} \mid (S_{i} - \widehat{S}) \mid \\ R^{2} = 1 - \frac{\sum _{i}(\widehat{S} - S_{i})^{2}}{\sum _{i}(\bar{S} - S_{i})^{2}} \end{array}\right. } \end{aligned}$$

## Results

### Trunk identification evaluation

**Fig. 10:**
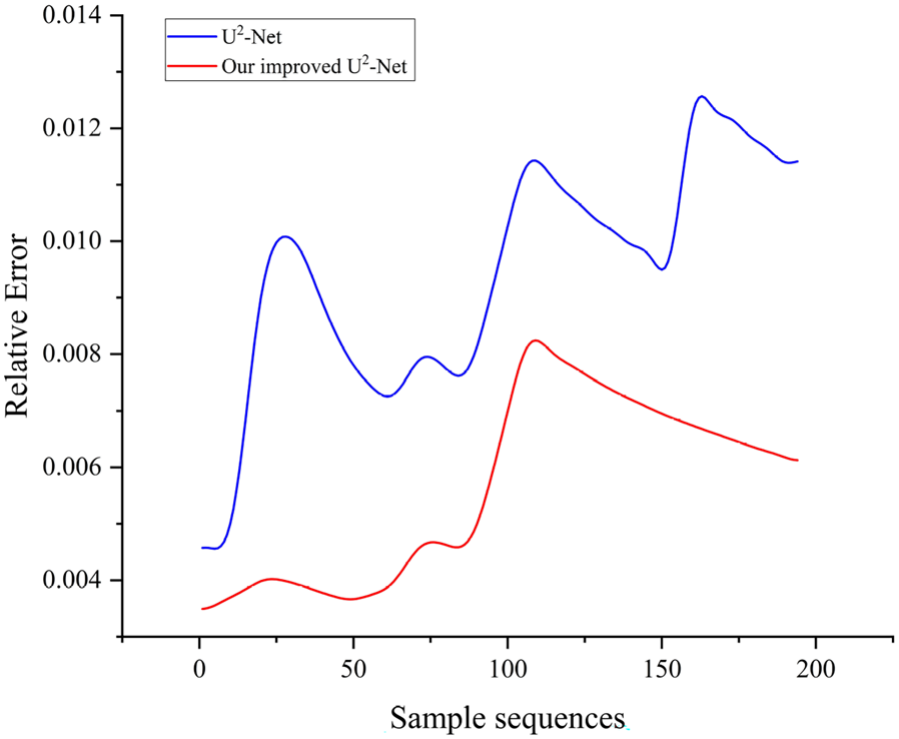
Comparison of the relative error of U$^{2}$-Net and our improved U$^{2}$-Net

In the accuracy evaluation results of model, U$^{2}$-Net results in *MAE* is ${0.99\times 10^{-2}}$, $S_{m}$ is 0.96, $F_{m}^\omega$ is 0.96, and mean $E_{m}$ is 0.97. Our improved model has *MAE* of $0.76\times 10^{-2}$, $S_{m}$ of 0.98, $F_{m}^\omega$ of 0.99, and mean $E_{m}$ of 0.99 (Fig. 10). Among the 526 samples obtained in the field measurements, only four samples in which occlusion affected the accuracy of trunk contour identification, further affecting the results of SD measurements (Fig. 10). Overall, most of the samples can be accurately identified, with only four images producing significant deviations of +23 pixels, -25 pixels, +27 pixels, and +24 pixels, respectively.

### Field measurements

**Fig. 11:**
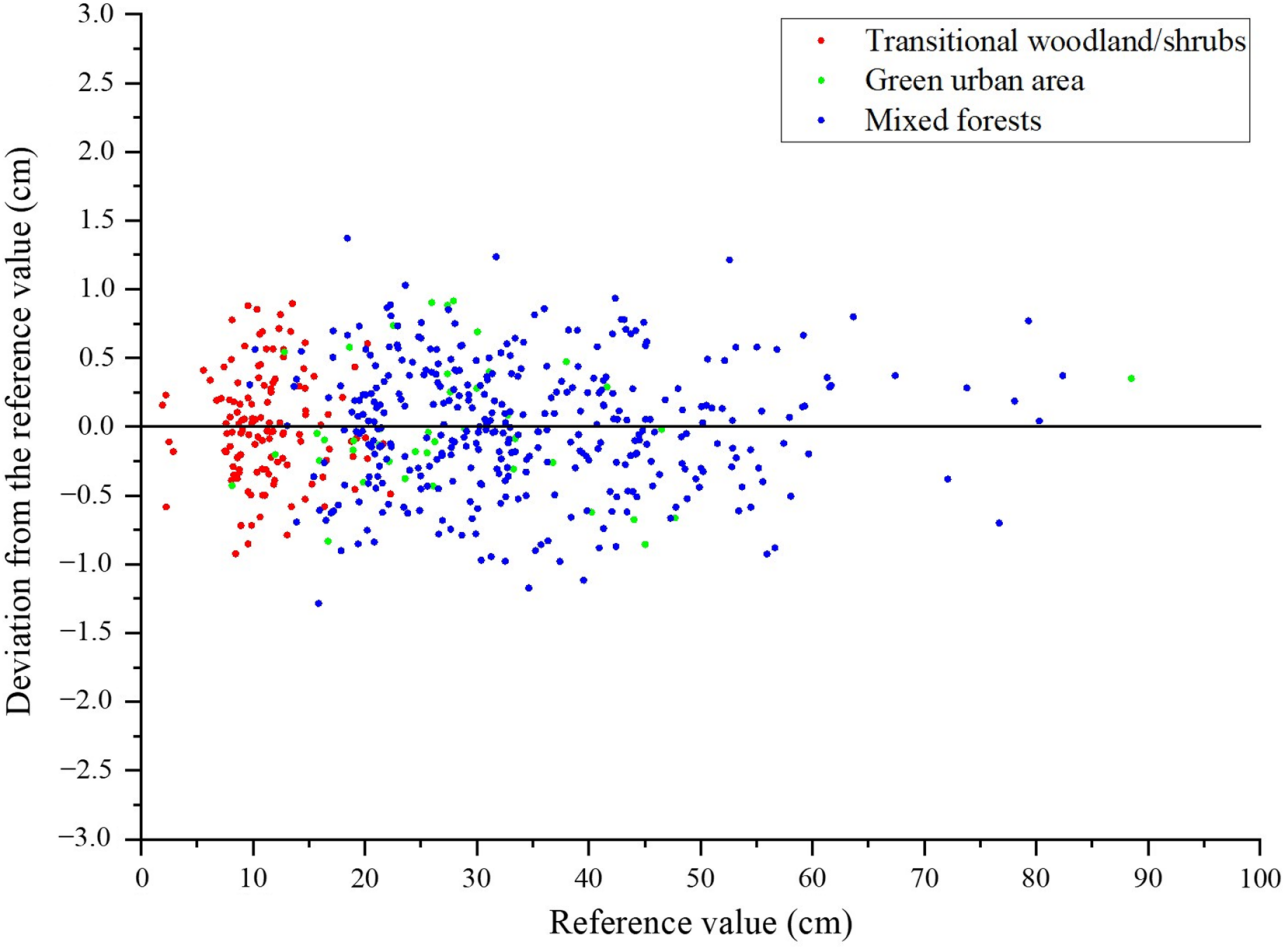
Deviation between the measured SD and the reference value measured by the tape measure

We compared the measurement deviations in different plots (Fig. 11, Table 2). The SD in the transition forest area/shrub ranged from 2.00 cm to 24.00 cm, with a *MAE* of 0.32 cm. Eight trees out of 129 had an absolute deviation greater than 0.80 cm and the minimum deviation was 0.11 cm. The SD in the mixed forest ranged from 10.00 cm to 82.00 cm, with *MAE* of 0.38 cm. The maximum absolute deviation was 1.34 cm out of 355 trees, with the minimum absolute deviation being 0.10 cm. The SD of one tree in the green urban area was 89.00 cm. The other trees ranged from 8.00 cm to 48.00 cm, with a *MAE* of 0.39 cm, with no significant difference.

**Fig. 12:**
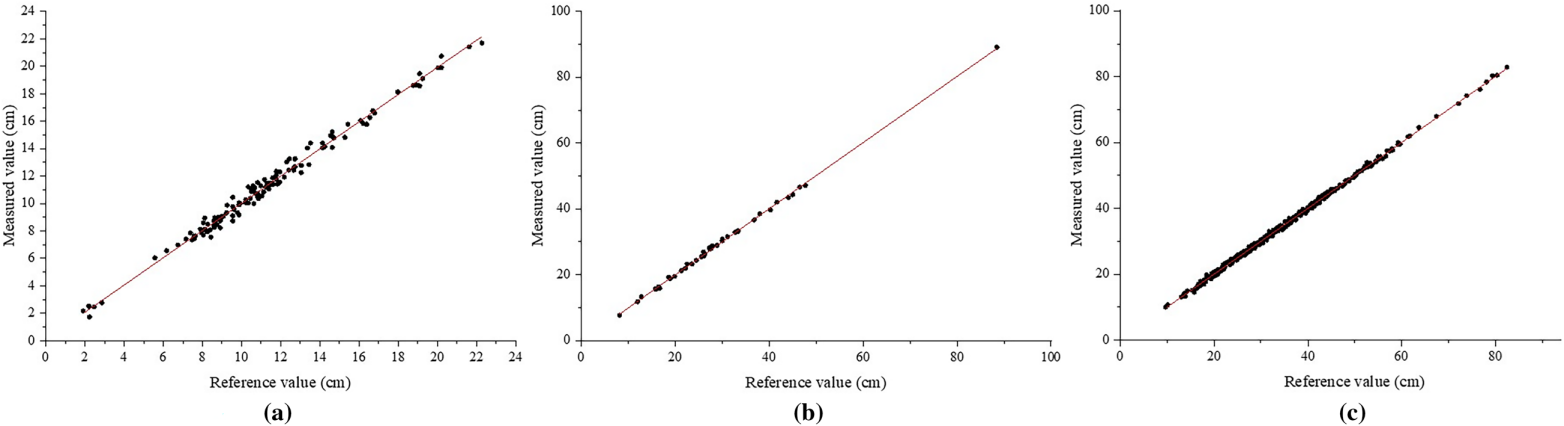
Comparison of the accuracy of SD measurements under different plots. **a** Transitional woodland/shrub. **b** Mixed forest. **c** Green urban area

To evaluate the correlation between the measured and reference values, linear regression and correlation analyses were done in different plots (Fig. 12). The results showed that the measured and reference values were significantly correlated. The *MAE*, *MSE*, *RMSE* and $R^{2}$ indicated that the measured values were close to the reference values (Table 2). We also excluded the four samples with significant differences and performed independent sample t-test on the overall sample. p was less than 0.01 at significance level equal to 0.05. There was no significant difference between the measured and reference values.

**Table 2:** Measurement accuracy at different measurement locations

	MAE (cm)	MSE (cm)	RMSE (cm)	$R^{2}$
Transitional woodland/shrub	0.32	0.16	0.40	0.9947
Mixed forest	0.38	0.21	0.46	0.9994
Green urban area	0.39	0.22	0.47	0.9993
All stems	0.36	0.20	0.45	0.9995

### Optimal spot size

**Fig. 13:**
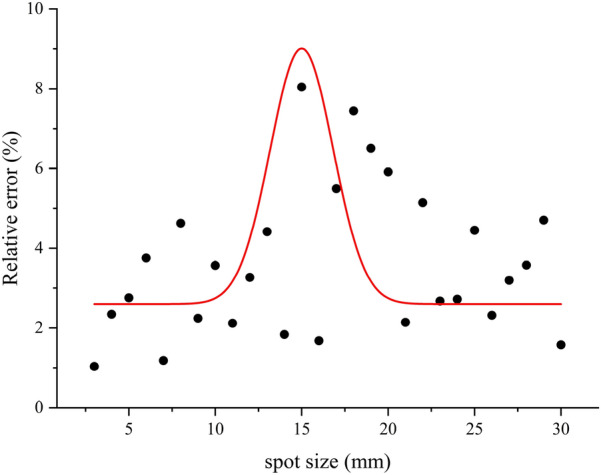
Effect of different spot sizes on the measurement accuracy of a standard cylinder with a diameter of 12 cm

To verify the effect of spot size on measurement accuracy, a standard cylinder of 12 cm diameter was tested using different spot sizes. We found that spot sizes of 3 mm and 30 mm resulted in the lowest relative error (Fig. 13). With this in mind, we use a 3 mm spot size laser module in transitional woodland/shrub. For mixed forest and green urban area, we use laser modules with the 30 mm spot size, as most trees are larger than 10 cm.

## Discussion

### Characteristics of the device

We developed a device based on image sensor and laser module to estimate SD of individual tree and evaluate the accuracy of measurements by comparing their corresponding field measurements. Different from the work of Fan et al. [49] and Song et al. [37], the collimated beam emitted by the laser module keeps the spot shape constant in natural scenes and can be used as a reference and anchor point. Image sensor can retain texture and shape features in images. This study evaluated the accuracy of the measurement method in different natural scenarios. The results show that our device can automatically locate the measurement position and measure SD with high accuracy.

Our device is designed not to preserve the color characteristics and spatial relationship characteristics of the image. Many image-based devices measure spatial relationships among trees and tree height in addition to DBH [36, 50, 51]. Some studies use the color features of the images for tree species recognition [52, 53]. In contrast to these studies, our device focuses on SD measurements.

### Influence of the natural scenarios on the method

The two key steps in measuring SD in the field are localizing and estimating the diameter. Fan et al. [49] determined the measurement position by manually obtaining the base point of the target tree, while Wu et al. [36] determined it by using the spatial relationship features of the image and the complex algorithm of coordinate system transformation. In transitional woodland/shrub, the spot with 3.00 mm diameter was adopted as a reference to calculate the SD. The measurement position was determined using the significant change in trunk contour width. For mixed forests and green urban areas, the measurement location was determined based on a linear relationship between spot diameter (30.00 mm) and measurement height. In addition, the spots with wavelengths of 680 nm and 980 nm were compared during the measurement. The spot with a wavelength of 980 nm could fit the perception of a digital camera. The spot with a wavelength of 680 nm was difficult to avoid the interference of direct sunlight.

Uneven lighting conditions on trees can lead to obvious errors in tree contour extraction. Only texture and shape features of the image are retained in the panchromatic images, which can well avoid this problem. In addition, bark surface grooves, unevenness, color differences, and ambient lighting conditions are all factors that affect the ability of the algorithm to extract salient image features. Our improved U$^{2}$-Net extracted the contour of the tree, the u-shaped structure could keep the output scale and input scale consistent. A-RSU module could overcome the influence of similar texture on model extraction (Fig. 10).

### The analysis of major error sources

As can be visually observed in Table 2, Figs. 11, 12, there is a strong relationship between the reference and measured values, where *MAE* is 3.60 mm and *RMSE* is 4.50 mm. In many previous studies, the results of SD calculations varied widely. Bayati et al. [54] performed 3D reconstruction of trees using near-earth photography combined with remote sensing and computer vision technology. The *RMSE* of measured DBH was 52.00 mm and relative bias was 0.28. Mulverhill et al. [55] obtained highly overlapping spherical images from different locations by two 12-megapixel cameras to generate high-quality point clouds for each target location. The *RMSE* of measured DBH was estimated to be 48.00 mm and relative bias to be 0.20. Ucar et al. [56] estimated the DBH of trees based on the iPhone X (Apple Inc.) and measurement app, and the average deviation is 3.60 mm at a distance of 1.50 m from the tree. Wu et al. [36] photographed multiple trees with smart phones and extracted tree contours by visual segmentation method. The measured DBH produced *RSME* of 2.17 mm and *MAE* of 15.10 mm. Song et al [37]. constructed an integrated device of digital camera and LiDAR and *RMSE* of measured DBH of 3.07 mm and bias of 0.06 mm. The accuracy of our method is consistent with most image-based measurement methods (Fig. 11).

The data in this study were obtained from natural scenarios, and 96.60% of the DBH values were between 2.00 cm and 60.00 cm. Among them, 0.01% had a deviation greater than 1.00 cm (Fig. 11). The error would not increase with increasing SD. The overall trend was consistent with many studies [36, 37, 57]. The uncertainty of the measurement error comes largely from the shading object. In addition, the diffraction of light and the angle of the beam to the trunk are also factors that affect the accuracy of the measurement.

The detection of target tree contour is also a crucial factor affecting the measurement accuracy. U$^{2}$-Net can process high resolution images with lower memory and computing costs to make the network deeper and provide excellent performance in complex environments. In our study, the U$^{2}$-Net can extract tree contours accurately. However, when it comes to processing semantic information, U$^{2}$-Net achieves sub-optimal performance. When there are branches, leaves or other shielding objects on the target tree, U$^{2}$-Net will have obvious object detection errors. In order to solve this problem, we added an *Attach* module to enhance its attention mechanism through the spot location of SDA. In addition, the number of training samples is also a factor affecting significant target detection. Although 1,600 images were used for training, the limited number of samples limited the detection capability of our improved U$^{2}$-Net. In the future, more data of different tree species should be collected and attention mechanism should be further developed.

### Application scenarios of the device

In the field measurement, factors such as weight and economic cost of the device are important factors in device selection. In this section, we compare the weight, economic cost, integration, and measurement range of the method and provide some suggestions for application scenarios.

*Weight of device.* The total weight of our device is 0.28 kg. The weight of LiDAR is between 0.20 kg and 15.00 kg. The weight of digital camera ranges from 0.30 kg to 0.80 kg. In previous work, the weight of the device of Ma et al. [58] is about 3.00 kg. The device of Song et al. [37] is only about 0.60 kg. The weight of our device is minimal relative to these devices, making it easy for foresters to measure and carry.

*Economic cost.* The cheapest measuring device used in nursery survey work is a tape measure, which usually sells for about 5.00 dollars. The price of LiDAR tends to be proportional to the measurement accuracy and range, usually above 23,000.00 dollars. The total cost of the device for Song et al. [37] was about 2,000.00 dollars. The economic cost of passive measurement methods using smartphones, on the other hand, is between about 300.00 dollars and 3,000.00 dollars [36, 58]. The hardware cost of our device is $104.00 and the core module is $59.00.

*Device integration.* The image sensor and laser module are both economical and integrated. The model size of U$^{2}$-Net is 4.70 MB, the inference speed is 40 FPS, and the SDA is only 0.50 MB. The method meets the conditions and limitations of running on a development board or Raspberry Pi. Further weight reduction and significant cost reduction will be achieved in the future with the mass production of the equipment.

*Measuring range.* The device is able to work under different lighting conditions and complex environments, and the working distance of 1.50 m to 10.00 m can ensure the accuracy of the device in measuring SD. For saplings, shrubs and multi-stemmed trees, close measurements are best. For measuring DBH of trees, measuring at a distance of 5.00 m from the target tree can ensure a balance of accuracy and efficiency.

*Application scenarios.* Our device reduces operational or computational errors and significantly improves measurement efficiency compared to conventional tape. Unlike LiDAR and other photogrammetric means, our device does not measure tree SD at the plot level. Based on the explicit purpose of the device development, our device is focused on SD measurements of trees in forests. It is worth mentioning that the device can be applied to similar forestry or agricultural scenarios, such as SD measurement of trees along urban roads [59].

### Limitations

Our method is not adapted to large scale measurement of SD of trees. Moreover, the measurement of stem diameter of irregular trees is a challenge, such as trees that lean or have roots above the ground. In addition, the effect of the physical properties of the laser on the measurement accuracy is a topic that deserves to be explored.

## Conclusion

In this paper, we presented a novel device and method to measure SD of trees in natural scenes based on deep learning and a low-cost laser module. We explored the generality and automation of the method in depth and compared it with conventional methods. Our method requires less human intervention and can perform automatic SD measurement without touching the trees. We proposed an algorithm for spot detection to provide reference information for SD calculations and improved the U$^{2}$-Net to more accurately determine the linear relationship between spot and trunk contour. The field measurement results showed that our method achieved acceptable accuracy with *MAE*, *MSE*, and *RMSE* of 0.36 cm, 0.20 cm, and 0.45 cm, respectively. However, some factors such as shading still limited the measurement accuracy of the method. In the future, we will explore more rigorous image-based measurement models and obtain more forest structure parameters.

## Data Availability

Part of the datasets will be made publicly available upon acceptance of the study, but not for commercial use. The authors can provide the full data if reasonably requested.

## Abbreviations

SD: Stem diameter
DBH: Diameter at breast height
3D: Three-dimension
2D: Two-dimension
LiDAR: Light detection and ranging
TLS: Terrestrial laser scanning
SDA: Spot detection algorithm
